# Emerging mechanisms of pyroptosis and its therapeutic strategy in cancer

**DOI:** 10.1038/s41420-022-01101-6

**Published:** 2022-07-27

**Authors:** Liqing Lu, Ye Zhang, Xuemei Tan, Yulia Merkher, Sergey Leonov, Li Zhu, Yalan Deng, Huajun zhang, Dandan Zhu, Yuying Tan, Ying Fu, Ting Liu, Yongheng Chen

**Affiliations:** 1 Department of Thoracic Surgery, Xiangya Hospital, Central South University, Changsha, 410008 People’s Republic of China; 2 Department of Oncology, NHC Key Laboratory of Cancer Proteomics & State Local Joint Engineering Laboratory for Anticancer Drugs, National Clinical Research Center for Geriatric Disorders, Xiangya Hospital, Central South University, 410008 Changsha, Hunan China; 3 Department of Gastroenterology, Xiangya Hospital, Central South University, Changsha, China; 4 School of Biological and Medical Physics, Moscow Institute of Physics and Technology, 141700 Dolgoprudny, Moscow Region Russia; 5 Institute of Cell Biophysics, Russian Academy of Sciences, 142290 Pushchino, Russia; 6 Shanghai Institute of Precision Medicine, Ninth People’s Hospital, Shanghai Jiao Tong University School of Medicine, 200125 Shanghai, China; 7 National Clinical Research Center for Geriatric Disorders, XiangYa Hospital, Central South University, 410008 Changsha, China

**Keywords:** Cancer, Immunology

## Abstract

Pyroptosis, a type of inflammatory programmed cell death, is triggered by caspase cleavage of gasdermin family proteins. Based on accumulating evidence, pyroptosis is closely associated with tumour development, but the molecular mechanism underlying pyroptosis activation and the signalling pathways regulated by pyroptosis remain unclear. In this review, we first briefly introduce the definition, morphological characteristics, and activation pathways of pyroptosis and the effect of pyroptosis on anticancer immunity. Then we review recent progress concerning the complex role of pyroptosis in various tumours. Importantly, we summarise various FDA-approved chemotherapy drugs or natural compounds that exerted antitumor properties by inducing pyroptosis of cancer cells. Moreover, we also focus on the current application of nanotechnology-induced pyroptosis in tumour therapy. In addition, some unsolved problems and potential future research directions are also raised.

## Facts

- Pyroptosis is inflammatory types of programmed cell death that depend on the membrane damaging GSDMs family proteins.
- A lot of inflammatory factors are released by pyroptotic cell death, which exert effect on tumorigenesis.
- Pyroptosis can be triggered by chemotherapy, natural compounds and nanomaterials.
- Pyroptosis can change the tumour immune microenvironment and trigger robust antitumour immune responses.
- Triggering tumour pyroptosis combined with immunotherapy holds great therapeutic potential for cancer treatment.

## Open questions

- What are the mechanisms of cell death dependent on pyroptosis?
- What are the key signals for pyroptosis to activate the immune response?
- How critical are GSDMs membrane pores for the release of inflammatory factors to modulate tumour immune responses?
- What are the key signals that initiate pyroptosis by chemotherapy or natural compounds?

## Introduction

Cancers are developing at high rates and becoming the most important public health burden due to their high incidence and high mortality [1]. Despite improvements in diagnosis and therapies, cancers tend to progress extremely aggressively with poor survival rates. Cell death plays a key role in maintaining the homoeostasis and development of multicellular organisms [2]. The main strategy of cancer therapy is to induce cancer cell death. To date, several cell death pathways have been discovered, including necrosis, apoptosis, autophagy, necroptosis, ferroptosis and pyroptosis, which are divided into two categories: accidental cell death (ACD) and programmed cell death (PCD) [2]. ACD is induced by exposure to severe physical, chemical or mechanical insults. Necrosis is the only type of ACD and in infectious and non-infectious diseases and cancers [3]. However, PCD depends on the molecular mechanism, suggesting that it is regulated by drug or genetic interventions. Apoptosis is a type of PCD that has been extensively studied in a variety of cancers [4]. Apoptosis is characterised by cytoplasmic shrinkage, chromatin condensation, nuclear fragmentation and the formation of apoptotic bodies that are swallowed by neighbouring cells with phagocytic activity, usually without triggering inflammation [2]. Unlike apoptosis, pyroptosis was recently identified as an inflammatory form of cell death characterised by cell swelling, pore formation in the plasma membrane and rupture of the plasma membrane, resulting in the release of intracellular contents, such as IL-1β and IL-18, and eventually inducing an inflammatory reaction [5]. Pyroptosis has long been presumed to depend on caspases [6]. Caspases are activated by bacterial infection and endogenous danger signals. Activated caspases cleave the gasdermin family proteins and release an N-terminal domain with perforating activity to trigger pyroptosis [5]. However, recent studies have shown that cellular pyroptosis occurs in a caspase-independent manner, and researchers have redefined pyroptosis as gasdermin-mediated inflammatory death [5, 7]. On the one hand, inflammation recruits and activates immune cells, which ultimately promotes tumour clearance. On the other hand, inflammation may contribute to tumour growth [8]. In addition, numerous studies have shown that pyroptosis is associated with anticancer immunity [9–11]. The roles of pyroptosis in cancer immunity and tumorigenesis have altered antitumour treatment strategies. In this review, we will summarise the role and mechanism of pyroptosis in cancer progression and discuss the potential therapeutic value of cancer treatments targeting pyroptosis.

## The morphological characteristics of pyroptosis

The term “pyroptosis” was primarily coined to describe proinflammatory programmed necrosis occurring in *Salmonella*-induced macrophages of Salmonella through a mechanism dependent on inflammatory caspases 1 [12]. However, recent studies have revealed that pyroptosis is also induced by other caspases, such as caspases 3, 7 and 8, in some cells other than those of the monocyte lineage [13]. The Nomenclature Committee on Cell Death (NCCD) redefined pyroptosis as programmed inflammatory death mediated by members of the gasdermin family of proteins in 2018 [2]. Morphologically, pyroptosis is accompanied by both characteristics of necroptosis and apoptosis. Similar to apoptosis, pyroptosis is characterised by DNA fragmentation, chromatin condensation, and activation of caspases 3 and 7. However, chromatin condensation is different from its apoptotic counterpart [2]. In addition, the nuclei of pyroptotic cells remain intact and show TUNEL-positive staining in the early stage, which is different from apoptosis [14, 15]. pyroptotic bodies have been observed in pyroptotic cells through time-lapse electron microscopy. Similar to apoptotic bodies, the diameter of pyroptotic bodies were also 1–5 µm. pyroptotic bodies could be divided into an intermediate class between classic apoptotic bodies and apoptopodia [16]. Some studies have shown that GSDM family proteins act as executor proteins in pyroptotic cells [5, 7]. The N-terminal domain of GSDM family proteins integrates with phosphatidylinositol phosphates, phosphatidylserine and cardiolipin in the cell membrane and then oligomerizes to form a large 10–15-nm inner diameter pore [17, 18], while another research group suggested that the pore size is 21 nm [19]. In addition, PARP cleavage occurs during NLRP3 and NLRC4 inflammasome activation [20], which suggests PARP cleavage is also associated with pyroptosis. Necroptosis is a regulated form of programmed cell death mediated by RIPK3 and its downstream substrate MLKL [21]. RIPK3 phosphorylates the executioner MLKL, leading to the formation of MLKL oligomers, which then shift to the plasma membrane and form selective pores [22]. Similar to necroptosis, pyroptotic cells are characterised by early permeabilization of the plasma membrane. Mechanistically, the two processes are mediated by oligomerization and translocation of the pore/channel-forming proteins to the plasma membrane, while the activation of MLKL and GSDM family proteins depends on phosphorylation and proteolytic cleavage, respectively [16]. The morphological differences and similarities between the different forms of cell death are listed in Table 1.

**Table 1:** Characteristics of similarities and differences in pyroptosis, apoptosis and necroptosis.

Characteristics	Pyroptosis	Apoptosis	Necroptosis
Programmed cell death	+	+	+
Membrane rupture	+	−	+
Selective channels	−	−	+
Nonselective pore formation	+	−	−
Membrane blebbing	+	+	−
Osmotic lysis	+	−	+
DNA damage	+	+	+
Organelle swelling	−	−	+
Chromatin condensation	+	+	−
Caspase-1/4/5/11 activation	+	−	−
Caspase-2/7/10 activation	−	+	−
Caspase-3/6/8/9 activation	+	+	−
EtBr staining	+	−	+
PI staining	+	−	+
7-AAD staining	+	−	+
Annexin V staining	+	+	+
TUNEL staining	+	+	+
Cell swelling	+	−	+
Cell shrink	−	+	−
Intact nucleus	+	−	+
Pyroptotic bodies	+	−	−
Apoptotic bodies	−	+	−
Necroptotic bodies	−	−	+
Inflammation	+	−	+
PARP cleavage	+	+	−
Gasdermin cleavage	+	−	−
ROCK1 cleavage	−	+	−
Phosphorylation of MLKL/RIP3	−	−	+

## Molecular mechanisms of pyroptosis

**Fig. 1:**
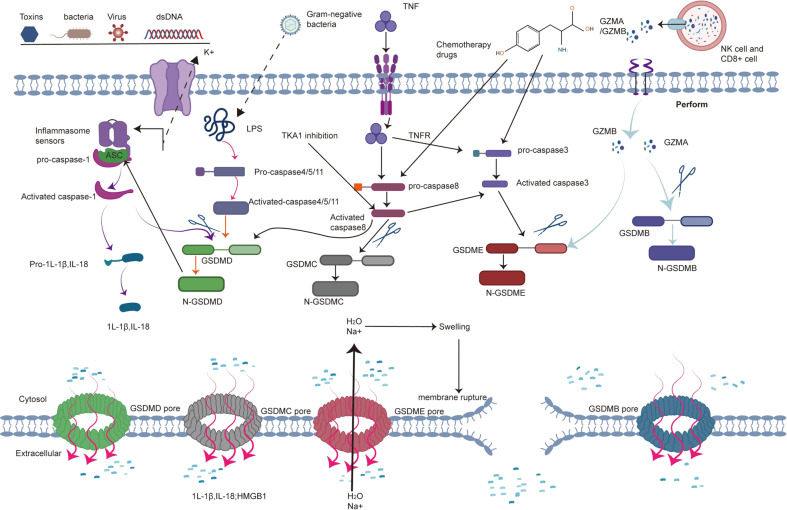
Molecular mechanism of pyroptosis. The canonical inflammasome is assembled from intracellular sensor proteins in response to PAMPs and DAMPs. Active caspase-1 cleaves pro-IL-1β and pro-IL-18 and results in the maturation of IL-1β and IL-18 that are subsequently released from the N-GSDMD pore. Active caspase-1 also cleaves GSDMD, releasing N-GSDMD to form nonselective pores on the plasma membrane, which allows the release of mature IL-1β and IL-18. In the noncanonical pathway, LPS directly binds to pro-caspase-4/5/11, resulting in activation of caspase-4/5/11, which cleaves GSDMD to trigger pyroptosis. In apoptotic caspase-induced pyroptosis, TNF activates caspase-8, which cleaves GSDMC and then induces pyroptosis in cancer cells. In addition, chemotherapeutic drugs trigger pyroptosis through the caspase-3/GSDME, caspase 1/GSDMD or caspase-8/GSDMC cascades. In the granzyme-A/B-dependent pyroptosis pathway, GzmA and GzmB from NK cells and CD8 + T cells enter cancer cells via perforin and recognise GSDMB and GSDME, respectively, to induce pyroptosis.

Inflammasomes induce pyroptosis through the canonical inflammasome pathway that depends on caspase-1 activation and the noncanonical inflammasome pathway through the activation of caspase-4/5/11 [5]. In addition, some studies have shown that proapoptotic caspase-3 activation also triggers pyroptosis by cleaving GSDME [23]. Historically, granzymes were generally considered to induce cell apoptosis through the activation of caspase-3 or its substrates [24]. However, recent studies have revealed that granzymes produced by natural killer cells trigger the pyroptosis of cancer cells in a caspase-independent manner [25, 26]. To date, four different pathways have been identified to induce pyroptosis. We will systematically describe the four pathways of pyroptosis induction in the subsequent sections (Fig. 1).

### Caspase-1-dependent classical pyroptosis pathway

To date, several inflammasomes have been confirmed, and the assembly of each inflammasome is determined by the activation of a unique pattern recognition receptor (PRR) in response to pathogen-associated molecular patterns (PAMPs) or danger-associated molecular patterns (DAMPs) in the host cell cytoplasm [13, 27]. Inflammasomes are multiprotein platforms consisting of three parts: (1) sensor proteins, including NLRP1, NLRP3, NLRC4, AIM2 and pyrin recognising PAMPs and DAMPs; (2) adapter proteins containing a caspase recruitment domain (CARD) and apoptosis-associated speck-like protein contains CARD (ASC) and (3) pro-caspase-1 [28]. The NLRP3 inflammasome is assembled upon the formation of a homotypic interaction between the amino-terminal pyrin domain (PYD) of NLRP3 and the PYD of ASC to recruit caspase-1 in response to bacterial, viral, and fungal pathogens [29]. Similar to NLRP3, AIM2 also promotes caspase-1 activation and regulates caspase-1-dependent maturation of IL-1beta and IL-18 in response to synthetic double-stranded DNA [30]. Pyrin is characterised by a PYD, two B-boxes and a coiled-coil structural domain. Unlike mouse pyrin, human pyrin contains a unique C-terminal SPRY/PRY domain that is mutated, increasing caspase-1 activation and IL-1β release [31]. Studies shown that activated caspase-1 cleaves the GSDMD protein at the middle linker (FLTD in humans and LLSD in the mouse), releasing a 31-kDa N-terminal fragment of GSDMD (GSDMD-N) that triggers pyroptosis by perforating the cell membrane and then forming nonselective pores, whereas the 22-kDa C-terminal fragment of GSDMD (GSDMD-C) inhibits the function of GSDMD-N [32, 33]. GSDMD-N alone triggers pyroptosis, while GSDMD-C and full-length GSDMD prevent cell death [33]. In addition, activated caspase-1 directly mediates the maturation of IL-1β and IL-18, which are released into the extracellular space through the pyroptotic pore [6].

### Caspase-4/-5/-11-dependent nonclassical pyroptosis pathway

Caspase-4/5/11 are activated by directly recognising cytosolic intracellular lipopolysaccharide (LPS) bound to the lipid A moiety [13]. Functional and chimeric studies further revealed that the CARD domain of caspase-11 interacts with lipid A [13]. Activated caspase-4/5/11 directly cleaves GSDMD into a C-terminal fragment and an N-terminal fragment [34]. The latter fragment forms pores in the member by lysing phosphoinositide- or cardiolipin-containing liposomes or on liposomes composed of natural polar lipid mixtures. In addition, activated caspase-4/5/11 do not mediate the maturation of pro-IL-1β/pro-IL-18 by direct cleavage, while they indirectly regulate cytokine maturation and release by modulating the NLRP3/caspase-1 pathways [5]. Notably, GSDMD-N also results in the efflux of K + , and eventually induces pyroptosis through a mechanism dependent on the activation of the NLRP3 inflammasome [35–37]. In addition, pannexin-1 and P2X7 were identified as vital proteins downstream of caspase-11-dependent pyroptosis induced in response to LPS [38].

### Pyroptosis induced by other caspases

According to several studies, apoptosis-related caspases other than caspase-1/11/4/5 also induce pyroptosis [39–41]. Caspase-3/8 were previously identified as the regulators of apoptosis. However, several recent studies have shown that TNF or chemotherapeutic drugs induce pyroptosis through caspase-3 cleavage of GSDME protein in GSDME-positive cancer cells or normal cells [39]. Furthermore, activated caspase-3 cleaved GSDME within the interdomain linker at site _267_DMPD_270_ in humans and _267_DMLD_270_ in mouse, liberating a GSDME-N fragment that possesses intrinsic pore-forming activity and then induces pyroptotic cell death [39]. Antibiotic chemotherapy drugs cause pyroptotic cell death in breast cancer cells through the caspase-8/GSDMC pathway [40]. In addition, Joseph Saran et al. also showed that activated caspase-8 during TAK1 inhibition also cleaves GSDMD and GSDME to induce pyroptosis [41].

### Granzyme-A/B-dependent pyroptosis pathway

Granzymes are a family of homologous serine proteases that are mainly expressed in CTLs and NK cells and cleave specific substrates in their target cells to induce programmed cell death [42]. To date, five human granzymes (granzymes A, B, H, K and M) and ten mouse granzymes (granzymes A, B, D, E, F, G, L, K, N and M) have been identified [43]. Granzyme A cleaves specific substrates after Arg or Lys basic residues and is also categorised as a “‘tryptase” [24]. Granzyme B is considered the main apoptosis-promoting granzyme because it cleaves target cell-substrate proteins at the same site as apoptotic caspases [42]. Granzyme-mediated cell death was previously generally presumed to be apoptosis. In 2020, Feng Shao et al. first found that cytotoxic lymphocytes mediate the death of GSDMB-expressing cells by inducing pyroptosis. Further mechanistic studies showed that lymphocyte-derived granzyme-A cleaved GSDMB at Lys[244] within the interdomain linker and released the N-terminal fragment to induce cell pyroptosis. Furthermore, interferon derived from activated cytotoxic lymphocytes increases the expression of the GSDMB transcript, which further contributes to GZMA- and GSDMB-mediated pyroptotic cell death [26]. In the same year, Judy Lieberman et al. also showed that granzyme B derived from cytotoxic lymphocytes directly cleaved GSDME after D270 to trigger caspase-independent pyroptosis in GSMDE-positive cells [25]. Thus, these two studies altered our view by showing that granzyme not only induces cell apoptosis but also induces caspase-independent pyroptosis in GSDME- or GSDMB-positive cells.

## Association between pyroptosis and cancer

Chronic inflammation may affect all stages of carcinogenesis. Long-term exposure to an inflammatory environment increases the risk of tumorigenesis [44, 45]. Pyroptosis is a form of lytic cell death that increases the release of mature IL-1 and IL-18, potentially influencing the pathogenesis of cancer [46]. In addition, pyroptosis mediates the inflammatory cell death of cancer cells and thereby suppresses cancer cell proliferation and migration. Therefore, pyroptosis plays a dual role in promoting and inhibiting tumorigenesis. We will summarise the role of pyroptosis in multiple tumours in the next sections.

### Pyroptosis and lung cancer

Chronic inflammation is one of the key factors contributing to the progression of lung cancer [47]. Researchers found higher GSDMD is the expression in NSCLC tissues than in matched adjacent nontumour tissues [48]. Patients with lung adenocarcinoma (LUAD), but not in squamous cell carcinoma (LUSC), presenting high GSDMD protein levels experience shorter survival, which indicates that GSDMD is an independent prognostic factor for LUAD [48]. Importantly, the authors further confirmed that activated NLRP3 inflammasome signalling triggers apoptosis instead of pyroptosis in GSDMD-deficient NSCLC cells [48]. In addition, Zhang et al. found that the tumour suppressor gene p53 directly interacts with NLPR3 to promote pyroptosis in non-small cell lung cancer [49]. LncRNA-XIST is overexpressed in NSCLC tissues and NSCLC cell lines compared to the corresponding control group. Silencing of lncRNA-XIST contributes to NSCLC cell pyroptosis by mediating NLRP3-Caspase-1 activation [50]. According to recent studies, pyroptosis is induced by ROS [51, 52]. In addition, silencing of lncRNA-XIST also promotes ROS production in NSCLC cells [50].

### Pyroptosis and liver cancer

Pyroptosis is also associated with the pathological process of liver cancer. Caspase-1 expression is downregulated in HCC tissues compared to adjacent normal tissues [53]. Lower levels of the DFNA5 protein are detected in hepatocellular carcinoma cells (HepG2) than in normal cells. Overexpression of DFNA5 in HepG2 cells inhibits cell proliferation [54]. The NLRP3 inflammasome is closely related to liver cancer progression. The NLRP3 inflammasome is downregulated in human HCC and inversely related to the tumour stage and pathological differentiation [55]. Hepatitis B virus X protein (HBx) is a significant factor causing HBV-induced hepatocellular carcinoma and increases GSDMD expression in normal liver cells exposed to H_2_O_2_ [56]. HBx initiates the pyroptosis of hepatic cells under hypoxia by mediating NLRP3 inflammasome activation [56]. Wei et al. showed that oestrogen represses HCC cell invasion and migration through the upregulation of the NLRP3 inflammasome [57]. This teams further showed that 17β-oestradiol (E2) represses HCC progression by triggering pyroptosis [58]. The long noncoding RNA SNHG7 is upregulated in HCC and contributes to NLRP3-dependent pyroptosis via the miR-34a/SIRT1 axis [59]. Thus, the inflammasome may be a potential therapeutic target for HCC.

### Pyroptosis and breast cancer

Based on accumulating evidence, GSDME functions as a tumour suppressor in many cancers. GSDME downregulation is associated with shorter survival of patients with breast cancer [25]. GSDME expression varies in different breast cancer cell lines. GSDME is expressed at higher levels in EMT6 triple-negative breast cancer and at lower levels in 4TIE triple-negative breast cancer [25]. Patients with breast cancer presenting higher levels of caspase-1, IL-1β and GSDMD exhibit an improved survival rate and lower histopathological grade and lymph node metastasis [60]. Mesenchymal stem cell (MSC)-based approaches are regarded as new cancer therapeutic strategies in many cancers due to their nourishing effect on the cancer microenvironment [61]. Yang Jiao et al. first found that factors secreted from human umbilical cord mesenchymal stem cells (hUCMSCs) trigger MCF7 cell pyroptosis. Furthermore, RNA-sequencing results show that the factors secreted from hUCMSCs significantly increase the expression of CASP4 and NLRP1 in MCF7 cells [62]. Recently, further studies elucidated that factors secreted from hUCMSCs induced MCF7 cell pyroptosis via the NLRP1-dependent canonical pathway and caspase-4-dependent noncanonical pathway [63]. In addition, factors secreted from hUCMSCs promote the interaction of NLRP1 with ASC to form inflammasomes, which are responsible for MCF7 cell pyroptosis [63].

### Pyroptosis and gastric cancer

Gastric cancer (GC) is one of the most common malignant tumours with a high incidence of relapse and metastasis in advanced stages [64]. In one study, GSDMA, GSDMC and GSDMD were silenced while GSDMB was overexpressed in GCs compared to normal tissues [65]. GSDMB is expressed at lower levels in normal gastric tissue samples than in the majority of precancer and cancerous samples, and the Alu element in the promoter region of GSDMB positively regulates GSDMB expression [66]. GSDMD is downregulated in GC compared to adjacent noncancerous tissues. GSDMD significantly inhibits the growth of tumours in vivo and in vitro. Furthermore, GSDMD represses the proliferation of GC cells by inhibiting the S to G2/M phase transition [67]. Although the potential mechanism of GSDMD in GC still requires further investigation, GSDMD is considered a target gene of chemotherapeutic drugs. GSDME functions as a tumour suppressor gene in gastric cancer [68]. In addition, Wang et al. showed that GC cells expressing GSDME at high levels undergo a switch from caspase-3-dependent apoptosis to pyroptosis in response to chemotherapeutic drug treatment [68]. Pyroptotic cell death is characterised by NLRP3 inflammasome activation [69]. NLRP3 plays a key role in the development of gastric cancer. Nia Sheng Ren et al. revealed that overexpression of the lncRNA ADAMTS9-AS2 promotes the pyroptotic cell death of GC cells by increasing the expression of NLRP3 [70].

### Pyroptosis and colorectal cancer

Colorectal cancer (CRC) is one of the most common malignancies, and its incidence and mortality rates are third among all cancers in the USA [47]. Chronic inflammation is one of the key pathogenic factors of colorectal cancer, implying that it is inflammation-related cancer [71]. Pyroptosis results in the release of inflammatory factors, which likely contribute to CRC development. Downregulation of GSDMC expression attenuates the proliferation of colorectal cancer cells, whereas overexpression of GSDMC contributes to its proliferation and tumorigenesis, implying that GSDMC has great potential as a therapeutic target for CRC [72]. GSDMD is expressed at significantly lower levels in human CRC tissues and negatively correlates with the prognosis of patients with CRC [73]. GSDME expression is significantly increased in the epithelial cells of the colonic mucosa of patients with inflammatory bowel diseases (IBD) compared to healthy humans. GSDME-mediated pyroptosis of epithelial cells enhance the progression of CRC by releasing HMGB1, which promotes tumour proliferation by activating the ERK1/2 signalling pathway [74]. According to another study, GSDME is expressed at higher levels in HT-29 and HCT116 cells but rarely expressed in SW480, CACO-2 and RKO cells [75]. Overexpression of miR-21-5p induces pyroptosis of CRC cells by downregulating the expression of TGFBI [76]. The lncRNA RP1-85F18.6 is upregulated and inhibits the pyroptosis of colorectal cancer cells [77]. GSDME-mediated pyroptosis of epithelial-cell release proinflammatory cytokines that contribute to progress of Crohn’s disease [78]. However, another research demonstrated that GSDME had no effect on intestinal cancer in the chemically or genetically induced intestinal cancer mouse model. Moreover, GSDME may only create an inflammatory microenvironment around the tumour [79].

In addition, some research groups have reported that interleukin 1β (IL-1β) and interleukin 18 (IL-18) can prevent the occurrence and progress of CRC, which suggests that the inflammatory cytokines can have both detrimental and beneficial effects depending on the context [80–82]. Inflammasomes are multiprotein complexes that can activate inflammatory caspases in response to the pathological signal. Inflammatory caspases lead to the production of inflammatory cytokines and the induction of pyroptosis. Pyroptosis can inhibit intracellular pathogens replication and trigger intracellular inflammatory response. Receptors that are able to assemble inflammasomes include leucine-rich repeat-containing proteins (NLR) family members (like NLRC4, NLRP1 and NLRP3) and proteins absent in melanoma 2 (AIM2). Cytokines activated by inflammasomes, especially IL-1β and IL-18, lead to inflammatory disorders. Studies demonstrated that the inflammatory disorders induced by high production of IL-1β and IL-18, plays a critical role in the pathogenesis of IBD and colorectal cancer [46, 83, 84].

### Pyroptosis and other cancers

GSDME is upregulation in the oesophageal squamous cell carcinoma (ESCC) compared to the normal tissue. STAT3β enhances chemosensitivity through resulting in the activation of caspase-3 and GSDME, and trigging cell pyroptosis in ESCC [85]. Wang et al. showed that miR-497 can downregulate PELP1 and eventually induce ESCC GSDMD mediated pyroptosis [86]. GSDMC is barely expressed in normal epithelial cells, but upregulated in malignant melanoma, which may be associated with melanoma invasion and metastasis [87]. It was shown that a combination of BRAF and MEK inhibitors treatment triggered the pyroptosis of human melanoma cells through inducing caspase-3 activation and GSDME cleavage [88]. HMGB1 upregulated was known to be negatively associated with melanoma survival [89]. Moreover, pyroptosis can release HMGB1 and enhance the progression of melanoma. Therefore, the role of pyroptosis in melanoma remains requires further study. Acute myeloid leukaemia (AML) is one of the most common hematopoietic malignancies. Zhou et al. reported that GSDMD can serve as a biomarker to estimate the sensitivity of curcumin in leukaemia treatment. Therefore, pyroptosis could be a potential novel manner for treating leukaemia [90].

GSDME is downregulated whereas GSDMD and GSDMC are upregulated in Ovarian Cancer [91]. LncRNA GAS5 induced the formation of inflammasome and caused pyroptosis in Ovarian Cancer [92]. Another research showed that lncRNA HOTTIP is upregulated in ovarian cancer tissues and cell lines, and downregulation of lncRNA HOTTIP could cause pyroptosis, preventing the progression of ovarian cancer [93]. GSDME was abundantly expressed in oral squamous cancer (OSCC), and GSDME expression was positively related to the prognosis of OSCC [94]. Some research further demonstrated that GSDME-mediated pyroptosis plays a key role in antitumor response [94, 95]. GSDMD was overexpression in glioma compared to nontumor tissues. Low expression of GSDMD was related to longer overall survival in glioma [96]. miR-214 represses glioma cells growth and metastasis by regulating the caspase-1-mediated cell pyroptosis [97].

The role of pyroptosis in a variety of tumours is summarised in Table 2.

**Table 2:** The role of pyroptosis in cancers.

Cancer types	The function of pyroptosis	References
Lung cancer	1. GSDMD promoted lung cancer development	[48, 49]
	2. P53 interacts with NLPR3 to promote pyroptosis and repress lung cancer development	
Liver cancer	1. DFNA5 inhibit HepG2 cell proliferation	[54, 55]
	2. The NLRP3 inflammasome is downregulated and represses HCC progression	
Breast cancer	1. GSDME and GSDMD repress breast cancer	[25, 60]
Gastric cancer	1. GSDMD and GSDME repress the gastric cancer cells proliferation	[67, 68]
Colorectal cancer	1. GSDMC promoted colorectal cancer development	[72–74]
	2. GSDMD inhibit colorectal cancer progression	
	3. GSDME-mediated pyroptosis of epithelial cells promote the progression of colorectal cancer	
Oesophageal squamous cell carcinoma	1. GSDME represses oesophageal squamous cell carcinoma progression	[85]
Melanoma cancer	1. GSDMC are upregulated and promote melanoma cancer progression	[87–89]
	2. BRAF and MEK inhibitors inhibit melanoma cancer progression via inducing pyroptosis	
	3. pyroptosis can release HMGB1 and enhance the progression of melanoma	
Acute myeloid leukaemia	1. GSDMD are upregulated and represses leukaemia progression	[90]
Ovarian cancer	1. GSDME is downregulated whereas GSDMD and GSDMC are upregulated and repress ovarian cancer progression	[91–93]
	2. lncRNA GAS5 and lncRNA HOTTIP represses ovarian cancer progression via inducing pyroptosis	
Oral squamous cancer	1. GSDME represses oral squamous cancer progression	[94]
Glioma	1. GSDMD represses glioma progression	[96, 97]
	2. Caspase-1-mediated glioma cell pyroptosis inhibit glioma progression	

## Effects of pyroptosis on cancer immunity

The mechanism by which the immune system recognises and kills tumour cells remains unclear. Immune cells recognise some DAMPs and then trigger a series of immune responses, including the activation of innate and adaptive immune cells [98]. IL-1 family members play key roles in host innate and adaptive immunity [99]. IL-1β and IL-18 are released into the tumour microenvironment through the pyroptotic pore to evoke an antitumour immune response [5, 9, 19]. Although various articles have described the key role of pyroptosis in cancer, the association between pyroptosis and anticancer immunity remains unclear.

In 2020, two studies first suggested that pyroptosis is tightly associated with antitumour immunity [25]. GSDME-expressing tumours increase the phagocytosis ability of macrophages and the numbers and functions of NK cells and CD8 + T lymphocytes [25]. In addition, natural killer cells induce the pyroptosis of GSDME-expressing tumour cells, and the underlying mechanisms are mediated by secreting granzyme B, which directly cleaves GSDME or activates caspase-3 to indirectly cleave GSDME [25]. Further animal experiments showed that tumour regression was abrogated in nude mice and T-cell-deficient mice, implying that the suppression of tumour growth by pyroptosis may be linked to the host immune system [25]. Feng Shao and colleagues reported that only ~15% of tumour cells undergo pyroptosis, which is sufficient to clear the entire tumour in animal experiments [100]. Likewise, pyroptotic tumour cell death is induced by NP–GSDMA3 and Phe-BF3 based on a bioorthogonal system, which increase the cytotoxic T cells and natural killer cell populations while decreasing the monocyte, neutrophil and myeloid-derived suppressor cell populations [100]. Based on these results, pyroptosis-induced tumour cells change the tumour immune microenvironment and trigger robust antitumour immune responses. Researchers also showed that pyroptosis-inducing approaches in combination with PD-L1 treatments more efficiently repress tumour growth than a single treatment by enhancing cancer immunity [100]. In addition, CD8 + T cells and NK cells also induce tumour cell pyroptosis through the granzyme-A/GSDMB axis [26]. GSDMB is expressed at higher levels in normal digestive tract epithelia, while GSDMB is usually silenced in digestive cancers. The authors showed that GSDMB, but not other GSDMs, is cleaved at Lys244 and induces target cell lytic death by granzyme A in NK cells [26]. PD-1 antibody treatment exclusively promotes the clearance of GSDMB^+^ tumour grafts [26]. Thus, the different molecular mechanisms of induced pyroptosis may depend on the variation in the GSDM protein level in target cell lines mediated by NK cells through perforin to deliver different granzymes.

CAR-T and CAR NK cell therapies, which are based on modifying autologous and allogeneic immune cells with chimeric antigen receptors (CARs) to target specific antigens of tumour cells, have attracted widespread attention as cancer treatments [101]. CAR T-cell therapy for cancer mainly induces caspase-3/GSDME-dependent tumour cell pyroptosis through the release of perforin and granzyme B [102]. Meanwhile, pyroptosis of tumour cells results in the activation of the caspase-1/GSDMD pathway in macrophages to release some proinflammatory cytokines, such as IL­6 and IL­1, and then triggers cytokine release syndrome (CRS) [102]. Recent papers suggest that the synergism of TNF and IFN-γ drives CRS through creating a positive feedback loop between inflammatory cell death and cytokine release [103–105]. Moreover, these two cytokines may be a significant molecular mechanism in the treatment of tumours through inducing pyroptosis, apoptosis, and necroptosis (PANoptosis) [106]. Furthermore, the quantity of perforin/granzyme B in CAR T cells but not existing CD8^+^ T cells induces GSDME-mediated target cell pyroptosis [102]. In addition, Chengui Lu and his team engineered a novel chimeric costimulatory transition receptor (CCCR) in CAR-T NK cells that consists of the extracellular region of PD-1, the transmembrane and intracellular regions of NKG2D, and the intracellular region of 41BB to convert the negative PD-1/PD-L1 signal to an activating signal and enhance the immunosuppressive efficiency of PD-1. Importantly, they elucidated that CCCR-NK92 cells eliminated the target tumour cells by inducing pyroptosis in PD-L1-positive H1299 cells [107].

**Fig. 2:**
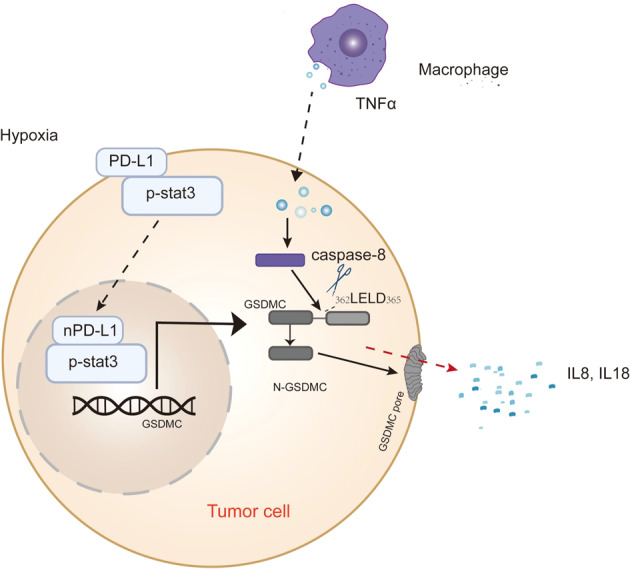
The mechanism of PD-L1 in pyroptosis. PD-L1 interacts with p-stat3 and then translocate into the nucleus to transcriptionally increase the expression of GSDMC, resulting in pyroptosis in response to hypoxic stress. TNFα in macrophages activates caspase-8, which cleaves GSDMC at the _362_LELD_365_ site, releasing N-GSDMC to induce the switch of apoptosis to pyroptosis in cancer cells.

PD-1 and PD-L1 are immune checkpoint regulators in many cancers [108]. Drugs targeting the PD-1/PD-L1 axis have emerged as effective cancer immunotherapy modalities that significantly prolong the survival of patients with multiple cancer types [109]. PD-L1 has been detected in the nucleus of circulating tumour cells or doxorubicin-treated breast cancer cells [110, 111]. Accumulating evidence suggests that PD-L1, which can be secreted extracellularly or translocated to the nucleus, plays a key role in the regulation of cancer immune evasion, tumorigenesis and immunotherapy [112]. However, the function of nuclear PD-L1 in tumour cells is unknown. Mien-Chie Hung et al. first reported that PD-L1 interacts with p-Y705-Stat3 and then induces PD-L1 nuclear translocation under hypoxic conditions [40]. The function of nuclear PD-L1 at the transcriptional level contributes to the expression of GSDMC, which then switched TNFα-induced apoptosis to pyroptosis [40]. Researchers further investigated the mechanism of nPD-L1 in hypoxia-mediated pyroptosis, and robust experiments proved that the PD-L1/p-Y705-Stat3 complex translocated into the nucleus and then induces pyroptosis via caspase-8/GSDMC in vivo and in vitro [40] (Fig. 2).

**Fig. 3:**
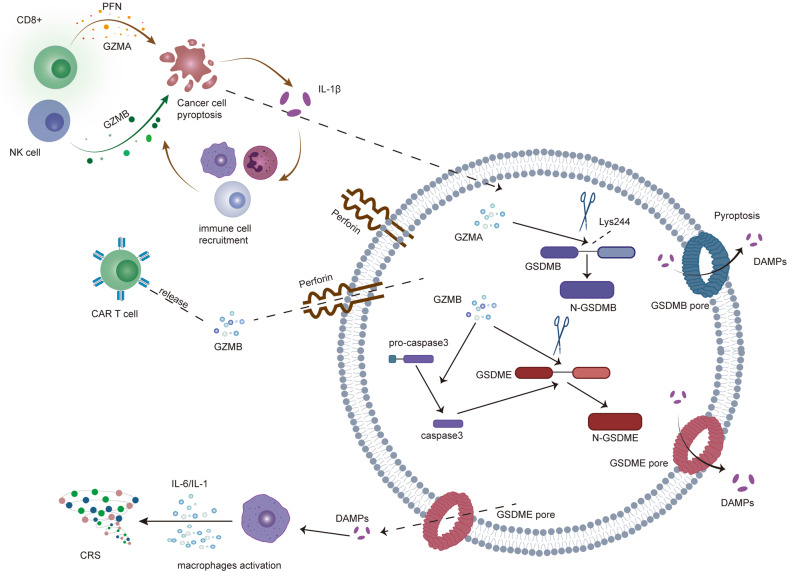
Positive feedback loops involved in pyroptosis and immune response. Pyroptotic cancer cells release a number of inflammatory factors, which in turn recruit immune cells and enhance the systemic immune response to kill cancer cells. CD4 + T cells, CD8 + T cells and CAR T cells secrete proteins of the granzyme family and perforin. Perforin forms membrane pores in tumour cells, through which granzymes translocate into tumour cells to trigger pyroptosis. Moreover, DAMPs released from the pyroptosis of cancer cells activate macrophages, which release large amounts of IL-6 and IL-1, inducing CRS. The positive feedback loop indicates that only a few cancer cells undergoing pyroptosis activate the immune system, alter the tumour microenvironment, and further trigger cell death.

From the abovementioned studies, we learned that pyroptosis is an immune-stimulated form of inflammatory cell death that can synergise with immune checkpoint drugs to improve the efficacy of tumour therapy. The crosstalk between pyroptosis and anticancer immunity is summarised in Fig. 3.

## Chemotherapy drugs and natural compounds induce pyroptosis in cancer

Chemotherapy is the standard treatment option for many types of cancer. However, despite the initial response to chemotherapeutic drugs, resistance inevitably occurs, and most patients die from chemotherapy-resistant cancers. Apoptosis is considered the primary form of chemotherapeutic drug-induced tumour cell death [113]. However, recent research has revealed that pyroptosis is a novel mechanism by which chemotherapy kills tumour cells [114]. Because a growing body of research has shown the key role of pyroptosis in cancer progression, treatments targeting pyroptosis represent a promising therapeutic strategy to treat cancer. Therefore, studies investigating the effects of chemotherapeutic drugs with a known ability to induce pyroptosis in patients with tumours are meaningful. Xuejun Sun et al. showed that chemotherapeutic drugs, such as 5-FU, switch caspase-3-dependent apoptosis to pyroptosis to inhibit the growth of gastric cancer cells with high GSDME expression [68]. Taxol treatment causes pyroptosis in nasopharyngeal carcinoma through a mechanism dependent on the activation of caspase-1 and cleavage of GSDMD. In addition, the inhibition of pyroptosis was presumed to be associated with taxol-resistant nasopharyngeal carcinoma [115]. Lobaplatin (chemical formula: C9H18N2O3Pt), a third-generation platinum anti-neoplastic drug, also causes GSDME-mediated pyroptosis in nasopharyngeal carcinoma by activating caspase-3 and regulating the proteasomal degradation of cIAP1/2 [116]. Moreover, lobaplatin induces caspase-3/GSDME-dependent pyroptosis in colorectal cancer cells. Knock out of GSDME converts lobaplatin-induced cell death from pyroptosis to apoptosis in HT-29 and HCT116 cells [51]. Cisplatin induces a higher rate of GSDME-mediated pyroptosis than paclitaxel in A549 cells, suggesting that cisplatin may be more effective at killing tumours with high levels of GSDME [117].

In addition to the chemotherapeutic drug-mediated pyroptosis of various cancer cells, some drugs and compounds derived from natural products also provoke pyroptotic cell death. For example, alpinumisoflavone, the main bioactive agent of *Derris Eriocarpa*, induces GSDME-dependent pyroptotic cell death in ESCC and HCC by activating caspase-3 [118, 119]. Berberine, a natural isoquinoline alkaloid, increases the expression of caspase-1 and induces pyroptosis to suppress the viability, invasion, and migration capacity of HepG2 cells in a dose-dependent manner [53]. In addition, Chen et al. showed that euxanthone, which is extracted from *Polygala caudata*, exerts anticancer effects on HCC by inducing pyroptotic cell death in a caspase-2-dependent manner [120]. Metformin also induces GSDME-dependent pyroptosis of ESCC by regulating the miR-497/PELP1 pathway [86]. Some traditional Chinese medicines or compounds inhibit the progression of NSCLC by inducing pyroptosis. For example, treatment with PPVI, a main saponin extracted from *Trillium tschonoskii* Maxim, induces A549 and H1299 cell death by switching apoptosis to pyroptosis and regulating NLRP3 inflammasome activation [121]. Other studies have documented that simvastatin, an antihyperlipidemic drug, induces pyroptosis in NSCLC cells by activating the NLRP3-caspase-1 pathway [122]. The L50377 piperlongumine (PL) analogue suppresses NSCLC cell growth by contributing to reactive oxygen species (ROS) generation and inducing apoptosis and pyroptosis. In addition, further studies revealed that L50377 causes pyroptosis in NSCLC cells by modulating ROS/NF-κB pathways [123]. Resibufogenin and Huaier extract also induce pyroptotic cell death in NSCLC cells [124, 125]. Treatment with C10, a novel 3’,5’-diprenylated chalcone, activates the upstream PKCδ/JNK pathway, which in turn activates caspase-3 and induces the cleavage of GSDME, leading to pyroptosis in prostate cancer [126].

Combination chemotherapy has become an alternative for cancer treatment and is widely used in clinical treatment due to the resistance of tumours to monochemical drug therapy. According to previous studies, gasdermin family proteins are expressed at low levels or absent in tumours due to DNA hypermethylation of promoters [25, 127]. A combined therapy consisting of DNA methylation inhibitors and chemotherapy triggers pyroptosis in tumour cells [127]. The combination of BI2536 and low-dose cisplatin synergistically reduces the viability of GSDME-positive oesophageal squamous cell carcinoma cells by inducing caspase-3-mediated pyroptosis [128]. AA and ATO synergistically inhibit colorectal cancer cell growth by activating apoptosis and pyroptosis [129]. BIX-01294 combined with cisplatin treatment induces pyroptosis through a mechanism dependent on autophagy via the Bax/caspase-3/GSDME pathway and enhances the chemosensitivity of NPC [130]. The combination of oxaliplatin and GW4064 synergistically suppresses the growth and colony formation of colorectal cancer cells. Further investigations indicated that GW4064 increase cell chemosensitivity to oxaliplatin by inducing BAX/caspase-3/GSDME-mediated pyroptosis [75]. Erkes et al. revealed that a BRAF-MEK inhibitor induces pyroptosis in melanoma cells through the cleavage of GSDME, increased intratumoural T-cell infiltration and immune responses, the release of proinflammatory factors and therapeutic effects [131]. Notably, the efficacy of a BRAF-MEK inhibitor in eliminating melanoma is completely blocked in immunodeficient GSDME-positive mice, implying that the potential molecular mechanism of BRAF-MEK inhibitor treatment of melanoma is a GSDEM-dependent antitumour immune response [131].

The agents that induce pyroptosis in a variety of tumours are summarised in Table 3.

**Table 3:** Summary of regents induce pyroptosis in cancers.

Cancer types	Regents	Experimental subjects	Target	References
NPC	BIX-01294 + cis-platinum	1. CNE-2Z cell; 2. xenograft animal model	Caspase-3/GSDME	[130]
	Taxol	1. HNE-2 and 5–8 F cells; 2. xenograft animal model	Caspase-1/GSDMD	[115]
	Lobaplatin	1. CNE-1, S26, HONE-1, SUNE-1 and CNE-2 cells	Caspase-3/GSDME	[116]
		2. xenograft animal model		
	Tanshinone IIA	1. HK1 cells	Caspase-1/GSDMD	[142]
GC	5-FU	1. SGC-7901 and MKN-45 cells	Caspase-3/GSDME	[68]
	BIX-01294 + Cisplatin	1. SGC-7901 cells; 2. xenograft animal model	Caspase-3/GSDME	[143]
ESCC	Alpinumisoflavone	1. KYSE510 and KYSE30 cell; 2. xenograft animal model	Caspase-3/GSDME	[118]
	BI2536 + Cisplatin	1. KYSE150 and KYSE510 cells; 2. Xenograft animal model	Caspase-3/GSDME	[128]
	Metformin	1. KYSE510 and KYSE140 cells; 2. xenograft animal model	GSDMD	[86]
HCC	Miltirone	1. HepG2 or Hepa1-6 cells; 2. HCC syngeneic model	Caspase-3/GSDME	[144]
	Berberine	1. HepG2 cell; 2. xenograft mouse model	Caspase-1	[53]
	Alpinumisoflavone	1. SMMC 7721 and Huh7 cells; 2. Xenograft HCC model	NLRP3 inflammasome	[119]
	Curcumin	1. HepG2 cell	GSDME	[145]
	E2	1. HepG2 cell	NLRP3 /Caspase-1	[58]
	Euxanthone	1. Hep3B and SMMC 7721 cells; 2. Xenograft HCC model	Caspase-2	[120]
	Sorafenib	1. Macrophages cell; 2. orthotopic HCC mouse models	Caspase -1	[146]
CRC	AA and ATO	1. SW620 and LOVO cells	Caspase -1	[129]
	GW4064 plus oxaliplatin	1. HT-29 and SW620 cells; 2. Xenograft mouse model	Caspase-3/GSDME	[75]
	Lobaplatin	1. HT-29 and HCT116 cells; 2. Xenograft mouse model	Caspase-3/GSDME	[51]
	Simvastatin	1. H1299 and A549 cells; 2. Xenograft mouse model	NLRP3/Caspase-1	[122]
NSCLC	PPVI	1. A549 and H1299 cells; 2. Xenograft mouse model	NLRP3/Caspase-1/GSDMD	**[**121]
	Cisplatin	1. A549 cell	Caspase-3/GSDME	[117]
	Paclitaxel	1. A549 cell	Caspase-3/GSDME	[117]
	L50377	1. A549 and NCI–H460 cells	GSDME	[123]
	Resibufogenin	1. A549 and H520 cells; 2. Xenograft mouse model	NLRP3 /Caspase-1	[124]
	Huaier extract	1. H520 and H358 cells; 2. Xenograft mouse model	NLRP3/Caspase-1	[125]
	Simvastatin	1. A549 and H1299 cells; 2. Xenograft mouse model	NLRP3/Caspase-1	[122]
BC	Omega-3 fatty acids	1. MDA-MB-231 cell	Caspase-1/GSDMD	[147]
	Dihydroartemisinin	1. MCF7 and MDA-MB-231 cells; 2. xenograft mouse model	AIM2/Caspase-3/GSDME	[148]
	Nobiletin	1. MCF7 and BT-549 cells; 2. xenograft mouse model	NLRP3/Caspase-1/GSDMD	[149]
	Tetraarsenic hexoxide	1. EO771, 4T1, Hs578T, and MDA-MB-231 cells; 2. Orthotopic mouse models	Caspase-3/GSDME	[150]
OSCC	Anthocyanin	1. Hacat, Tca8113 and SCC15 cells	NLRP3/CASPASE-1/GSDMD	[151]
CC	Tanshinone IIA	1. HeLa cell	GSDMD	[152]
	PCB29-pQ	1. HeLa cell	Caspase-1/GSDMD	[153]

*NPC* nasopharyngeal carcinoma, *GC* gastric cancer, *ESCC* oesophageal squamous cell carcinoma, *HCC* hepatocellular carcinoma, *CRC* colorectal cancer, *NSCLC* non-small cell lung cancer, *BC* breast cancer, *OSCC* oral squamous cell carcinoma, *CC* cervical cancer.

## Pyroptosis and nanomaterials

At present, drug combinations have achieved excellent efficacy in clinical cancer treatment, but the main challenges are to improve the bioavailability and minimise the side effects due to the low solubility and nontargeted properties of drugs. Due to the accelerated progress achieved in nanotechnology in recent years, it represents a possible approach to increase the therapeutic effect and decrease the toxicity of chemotherapy [132]. Nanotechnology, a controlled-release chemotherapy drug carrier, directly delivers chemotherapeutic drugs to targeted cancer cells while decreasing their accumulation in normal cells and tissues and reducing the side effects of chemotherapy and has been widely used in cancer treatment [132, 133]. Hence, the combination of nanotechnology with pyroptosis to cure cancer has become a hot research topic.

LipoDDP is a tumour-targeted nanoliposome carrying cisplatin that takes advantage of liposomes and activates caspase-3-mediated pyroptosis [127]. DAC is a well-known DNA methyltransferase (DNMT) inhibitor that inhibits *DFNA5* gene methylation and rescues GSDME protein levels in cancer cells when administered at a low dose [127]. LipoDDP is quite stable in vivo and shows excellent biocompatibility, a long blood circulation time, high drug loading efficiency, and other properties. The cotreatment of DAC with LipoDDP successfully initiates the immunological response of living systems and upregulates the relevant genes in various cell death pathways. Treatment with DAC and LipoDDP not only significantly suppresses tumour proliferation but also efficiently suppresses tumour metastasis. This combined therapy shows good antitumour properties and induces an immunological response, suggesting that is a promising option for tumour immunotherapy [127].

As a method to overcome the problem of a lower As_2_O_3_ concentration inside the tumour, As_2_O_3_-NP, a triblock copolymer of mPEG-PLGA-PLL, was employed to load As_2_O_3_, which increased GSDME-N expression and directly delivered As_2_O_3_ into HCC cells and then induced pyroptotic cell death [134]. Higher CXCR4 levels are associated with drug resistance of colorectal cancer. T22-DITOX-H6, a toxin-based nanoparticle, selectively targets CXCR4 + cells and induces caspase-11/NLRP3-mediated pyroptosis in vivo [135]. Therefore, this novel nanotechnology possesses great capacities to overcome tumour chemotherapeutic resistance.

A bioorthogonal chemical system was used to investigate the antitumour immune mechanism of pyroptosis. A nanobioorthogonal chemical system in which gasdermin A3 (GSDMA3) is combined with nanoparticles by the triethylsilyl (TES) ether linker releases activated GSDMA3, which binds to the cellular membrane and induces pyroptosis [100]. SLR20 is an agonist of retinoic acid-inducible gene I (RIG-I), another pattern recognition receptor (PRR) of the innate immune system [136]. Elion et al. described that SLR20 NP treatment inhibits tumour growth and metastasis by inducing pyroptosis and intrinsic apoptosis in breast cancer [137]. The constructed nanocomplex LP-R/C@AC eliminates gastric cancer cell growth by inducing pyroptosis [138]. Therefore, with the development of nanotechnology, we can apply nanomaterials as carriers for chemotherapeutic drugs or pyroptosis-related recombinant proteins to trigger pyroptosis and reduce the toxic effects of chemotherapy drugs.

## Conclusions and perspectives

Emerging evidence showed PCDs, including ferroptosis, necroptosis and pyroptosis, is highly related to the immune response, which have important functions in the regulation of cancer progression. Therefore, the induction of nonapoptotic cell death is a promising strategy for cancer treatment. For example, pyroptosis can trigger a strong immunostimulatory response in tumour microenvironment and increase cancer immunotherapy efficacy. Particularly, pyroptosis induces the upregulation of the gasdermin protein, then modulates M1 macrophages and T lymphocytes infiltration to increase cell sensitivity to anti-PD-1 mAbs [139–141].

As exploration revealed, apoptosis, necroptosis, pyroptosis and other types of PCDs, are tightly connected and cross-linked with each other. The cross-regulation between apoptosis, necroptosis, and pyroptosis indicates the presence of bridges between these pathways to coordinate cell death. Apoptosis is less immunogenic than necroptosis or pyroptosis, but it promotes proinflammatory cytokine release under a specific state. Hence, cell death pathways are not individuals working independently. Instead, these pathways are flexible molecular tools that produce a wide range of results and emit signals with a full spectrum of anti-inflammatory or proinflammatory molecules. The “death signal” is probably not an event that happens at a specific time, but the result of the delicate balance between pro-death and anti-death signal. Different key participants can break the fragile balance of cell environment from birth to death, or from proinflammatory to anti-inflammatory. The research on the complex regulatory mechanism of cell death will provide a theoretical basis for developing new drugs, and determine which therapy is appropriate in cancer treatment, either enhancement or prevention of cell death [139–141].

GSDME mRNA hypermethylation causes the low expression of its protein, thus suppressing the induction of pyroptosis in the majority of tumour cells. Demethylating drug treatment upregulates GSDME expression to enhance chemotherapeutic drug sensitivity and reduce drug resistance. Thus, other undefined gasdermin family members may also suppress tumour growth and provide novel directions for cancer therapies. In the tumour microenvironment, systemic immune responses are downregulated by immune suppressor cells. Pyroptosis releases proinflammatory intracellular contents and thus can be used for tumour immunotherapy. Cancer studies on pyroptosis are still in an early stage. By obtaining deeper insights into its mechanism, pyroptosis possesses great potential in cancer diagnosis and treatment.

With the rapid development of medical research technology, chemotherapeutic drugs, natural compounds and nanomaterials have been explored to trigger pyroptosis in cancer research. The amounts of nanomaterials increase our work productivity and quality of life. Due to the good merits of nanomaterials, stimulation in response to the tumour microenvironment is considered a new therapeutic advantage in specifically killing cancer cells through pyroptosis. Nanoparticles can selectively activate pyroptosis in cancer cells and deliver the corresponding compound or drug at the precise site in solid tumours, thereby substantially avoiding damage to normal tissues. However, nanomaterials are frequently used, which pose a great threat to our health. For example, MSNs are widely employed but cause liver injury induced by pyroptosis of normal liver cells. ITO nanoparticles result in a high mortality rate related to occupational diseases, such as pulmonary alveolar proteinosis or interstitial lung disease. In addition, AgNP15 induces ATF-6 degradation in normal cells, then activates the NLRP3 inflammasome, and eventually triggers pyroptosis.

Targeting pyroptosis is still at the preliminary exploration stage in biomedical fields, and many key problems must be solved to further strengthen the clinical transformation. In the future, five aspects should be taken into consideration mainly: (1) Novel nanomaterials should be designed to achieve more efficient synergistic pyroptosis anticancer therapies, and the physicochemical properties of the nanomaterials should be optimised, such as the size, shape, stiffness, chemical composition and surface charge, and we should determine the effect of nanomaterials on pyroptosis induction. (2) Nanomaterial specificity in inducing pyroptosis needs more preclinical and clinical experiments to clarify the molecular mechanism and control its potential side effects. (3) Cellular function alterations caused by pyroptosis and signalling pathway alterations mediated by nanomaterial must be evaluated. (4) The combination of pyroptosis and other synergistic pathways should be explored, and the crosslink between pyroptosis and other pathways can be exploited for the best tumour treatment strategy. (5) To achieve clinical translation, we need more detailed mechanistic insights, as well as real-time imaging studies, to evaluate the utility of pyroptosis in combating apoptosis-resistant cancers. Thus, the rational use of nanomaterials is important. Building a powerful toxicity database will provide an indispensable clinical basis and protect patients from the side effects of nanomaterials. Hence, in the near future, we will anticipate high-efficacy nanoparticles to show great potential in pyroptosis-based cancer therapy.

## Data Availability

Data sharing not applicable to this article as no datasets were generated or analysed during this study.
